# Neurodegeneration of the mastoid segment of the human facial nerve associated with senescence

**DOI:** 10.1016/j.bjorl.2026.101784

**Published:** 2026-03-19

**Authors:** Ivam Luiz Mariano Rocha, Joel Lavinsky, Mateus Belmonte Macedo, Adriana Ubirajara Silva Petry, Taís Malysz

**Affiliations:** a Universidade Federal do Rio Grande do Sul (UFRGS), Instituto de Ciências Básicas da Saúde (ICBS), Postgraduate Program in Neuroscience, Porto Alegre, RS, Brazil; b Universidade Federal do Rio Grande do Sul (UFRGS), Instituto de Ciências Básicas da Saúde (ICBS), Department of Morphological Sciences, Porto Alegre, RS, Brazil; c Technical Director of the Death Verification Service of the Vila Nova Hospital Association, Porto Alegre, RS, Brazil

**Keywords:** Facial nerve, Morphometry, Aging, Neurodegeneration, Myelinated fibers

## Abstract

**Objective:**

To describe and analyze the microscopic morphometry of the mastoid segment of the human facial nerve in the region of the stylomastoid foramen, in different age groups of elderly individuals.

**Methods:**

Eighteen blocks of temporal bones from cadavers aged between 63 and 94 years old were used and divided into 3 age groups: 60–70, 71–80 and over 80-years-old. The nerve was collected, fixed in a glutaraldehyde and paraformaldehyde solution and then subjected to dehydration and resin embedding processes. Semi-thin cross-sectional sections (1 μm) were obtained using an ultramicrotome and stained with toluidine blue. Histological images were captured and analyzed using Zen 2.6 software. The variables were described by mean and standard deviation and compared between age groups (SPSS; p < 0.05).

**Results:**

The Cross-Sectional Area (CSA) of the nerve, diameter of the myelinated fiber, CSA of the myelinated fiber, thickness of the myelin sheath and percentage of total area occupied by myelinated fibers were lower in the groups with older ages, with significant differences among all age groups (p < 0.001). The total number of myelinated fibers and density remained constant and did not present significant differences between age groups. CSA and axon diameter decreased at older ages, with significant differences only between the second and third groups (p < 0.001). The degree of myelination (g ratio) and the percentage of area occupied by the endoneurium, unmyelinated fibers and degenerating tissue were higher in the older age groups (p < 0.001). In the older age groups, there was an increase in the percentage of myelinated fibers with lower CSA and a reduction in the percentage of myelinated fibers with higher CSA.

**Conclusion:**

The results indicate that the facial nerve presents morphometric variations suggestive of neurodegeneration, associated with aging.

**Level of evidence:**

Level V.

## Highlights

• There was a progressive reduction in myelination of the facial nerve with aging.
• Reduction in the cross-sectional area of facial nerve axons occurs at older ages.
• Increase in the quantity of small myelin fibers at older ages.
• Decrease in the quantity of large myelin fibers at older ages.
• The number and density of nerve fibers were not altered during aging.

## Introduction

The facial nerve, the seventh cranial nerve, is considered mixed because it contains motor, sensory and parasympathetic fibers. It originates from the bulbopontine sulcus, from where a motor root emerges that innervates muscles of facial expression and an intermediate nerve that carries special sensory fibers for taste, somatic sensory fibers and parasympathetic visceral motor fibers.^1^

Aging can lead to microstructural changes in nerve fibers, which can compromise the functioning of the Peripheral Nervous System (PNS). Research indicates that is associated with increased demyelination, brain atrophy, and motor and sensory impairment.^2^, ^3^, ^4^ Older individuals are more vulnerable to axonal injury and have a lower capacity for remyelination compared to younger individuals.^5^^,^^6^ This highlights the importance of the myelin sheath and its role in maintaining efficient nerve conduction and, consequently, the motor and sensory functions of the fibers.^3^ In addition, the diameter of the nerve fiber and the degree of myelination are closely linked to the degree of neural regeneration.^7^, ^8^, ^9^

Unraveling the changes in the architecture of the facial nerve with age, including possible microanatomical variations in its path, is essential for the development of more effective surgical techniques, preventing iatrogenic injuries and also contributing to the understanding of how the process of neurodegeneration occurs in the PNS and its associated pathologies.^10^^,^^11^

This study analyzes the micromorphometric changes of the facial nerve and correlating them with age groups. These findings provide valuable information that helps in understanding the clinical and surgical implications related to morphological changes of the facial nerve.

## Methods

**Fig. 1:**
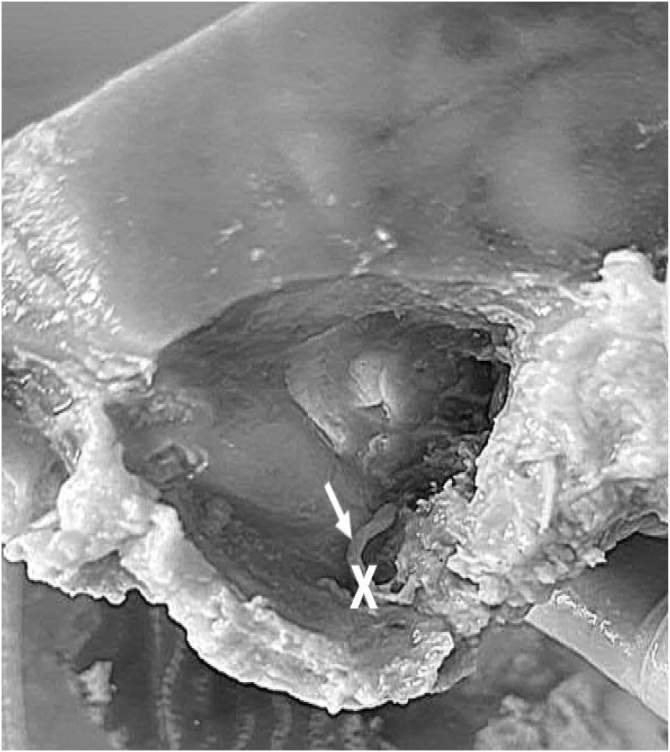
Image showing the right temporal bone of a 69-year-old individual, after mastoidectomy and removal of the fallopian canal of the facial nerve. The arrow shows a facial nerve with its histological structure preserved. In addition, we can see in “X” the site where a portion of approximately 3 mm of the mastoid segment of the facial nerve is collected, located distally to the chorda tympani nerve and immediately proximal to its passage through the stylomastoid foramen.

To this study 18 facial nerves obtained by anatomical pieces of human temporal bones extracted from human cadavers (10 men and 8 women) provided by the donation collection of the Human Anatomy Laboratory of the Federal University of Rio Grande do Sul (UFRGS). The research Project was registered and approved at UFRGS with number 43748. The inclusion criteria were age over 60-years, preserved temporal bone structure and preserved microscopic structure of the facial nerve to enable analysis. Access to the temporal bone was achieved by mastoidectomy and removal of the fallopian canal of the facial nerve in the right temporal bone of the cadavers. A portion of approximately 3 mm of the mastoid segment of the facial nerve was collected, located distally to the chorda tympani nerve and immediately proximal to its passage through the stylomastoid foramen (Fig. 1). The samples were organized into three distinct age groups: 60–70 years (n = 9), 71–80 years (n = 4) and over 80-years (n = 5).

The nerve was fixed in a solution containing 2.5% glutaraldehyde and 4% paraformaldehyde in 0.1 M phosphate buffer for 48 h. Subsequently, the material was washed, followed by post-fixation in 1% osmium. The material was then dehydrated in increasing concentrations of acetone, progressively embedded in Durcupan ACM resin (Fluka, Switzerland), and placed in an oven at 60 °C for polymerization. Semi-thin sections (900 nm) of the nerve segments were obtained in an ultramicrotome (Leica Ultracut UCT 2.0, Germany). The slides containing the sections were stained with 1% toluidine blue.

**Fig. 2:**
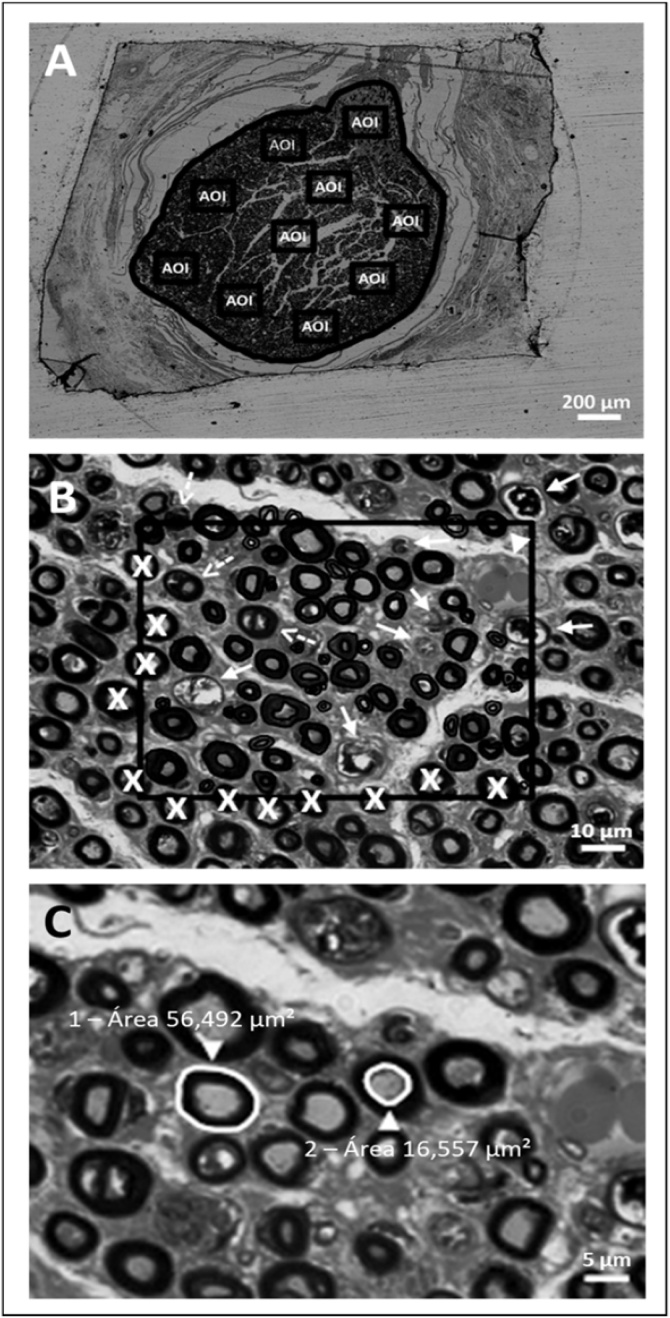
Images demonstrating the planar morphometry protocol used. Image “A” shows a cross-section of the mastoid segment of the human facial nerve, demonstrating the allocation of the 10 areas of interest (AOI) and the respective delimitation of the intraperineural area at a final magnification of 50×. In “B”, the method of selection and measurement of nerve fibers is represented, demonstrated from the delineation of the axonal area and the area of the myelinated nerve fibers that presented preserved cytoarchitecture at a final magnification of 1,000×. The dotted arrows indicate nerve fibers in early stages of neurodegeneration, while the other arrows indicate more advanced stages. The arrowhead indicates the presence of a macrophage. In addition, it can be observed that the fibers containing an “x” were not measured because they touched the AOI exclusion bar. In “C”, the upper right quadrant of the AOI shown in “B” is enlarged to highlight the delineation performed to obtain the value of the measurement of the area of a myelinated fiber (1). The measurement of the axonal area of a fiber with the respective value obtained is also exemplified (2).

From each nerve, a cross-sectional section (900 nm) was selected to calculate the Cross-Sectional Area (CSA) using a 5× objective (Fig. 2A) and 10 images of rectangular areas of interest (AOI 90 × 70 μm) were sequentially obtained at 100× magnification (Fig. 2B) using a Nikon Eclipse E-600 microscope, equipped with a high-performance charge-coupled device camera. In each image captured of the nerve, 10 Areas Of Interest (AOI) with 6300 μm^2^ each were inserted. All fibers within and those that come into contact with the upper and right lateral margin of the AOI were analyzed (Fig. 2B). In total, 36003 fibers were analyzed, and 198 images collected. The images were analyzed using the Zen 2.6 software (Blue edition) based in previous morphometrical studies.^12^, ^13^, ^14^ The microscopic morphometric data analyzed were: nerve cross-sectional area, estimated total number and density of myelinated nerve fibers, mean cross-sectional area of axons and myelinated fibers, mean diameter of axons and myelinated fibers, mean thickness of myelin sheath, degree of myelination (g ratio), percentage of total area occupied by myelinated fibers and the percentage of area occupied by endoneurium, unmyelinated fibers and degenerating tissue.

To obtain axonal diameter values, the axonal area of each fiber was measured, and the value obtained was converted to the diameter of a circle with equivalent area by the formula 2√(A/π). The diameter of the myelinated fiber, including axon and myelin sheath, was calculated using the same formula (Fig. 2C). To calculate the thickness of the myelin sheath, it was necessary to subtract the estimated diameter of the axon (d) from that of the entire nerve fiber (D) by the formula (D-d)/2 modified from Ramkumar et al.^13^ The fibers were classified according to their area as small (< 10 μm^2^), medium (10–20 μm^2^) and large (>20 μm^2^).^10^ The g ratio was calculated from the ratio between the internal axonal diameter (d) and the total external diameter (D) according to the formula g = d/D.^14^

The variables were described by mean and standard deviation and compared between age groups using Analysis of Variance (ANOVA) complemented by the Tukey test. The significance level adopted was 5% (p < 0.05) and the analyses were performed using the Statistical Package for the Social Sciences (SPSS) version 27.0.

## Results

**Fig. 3:**
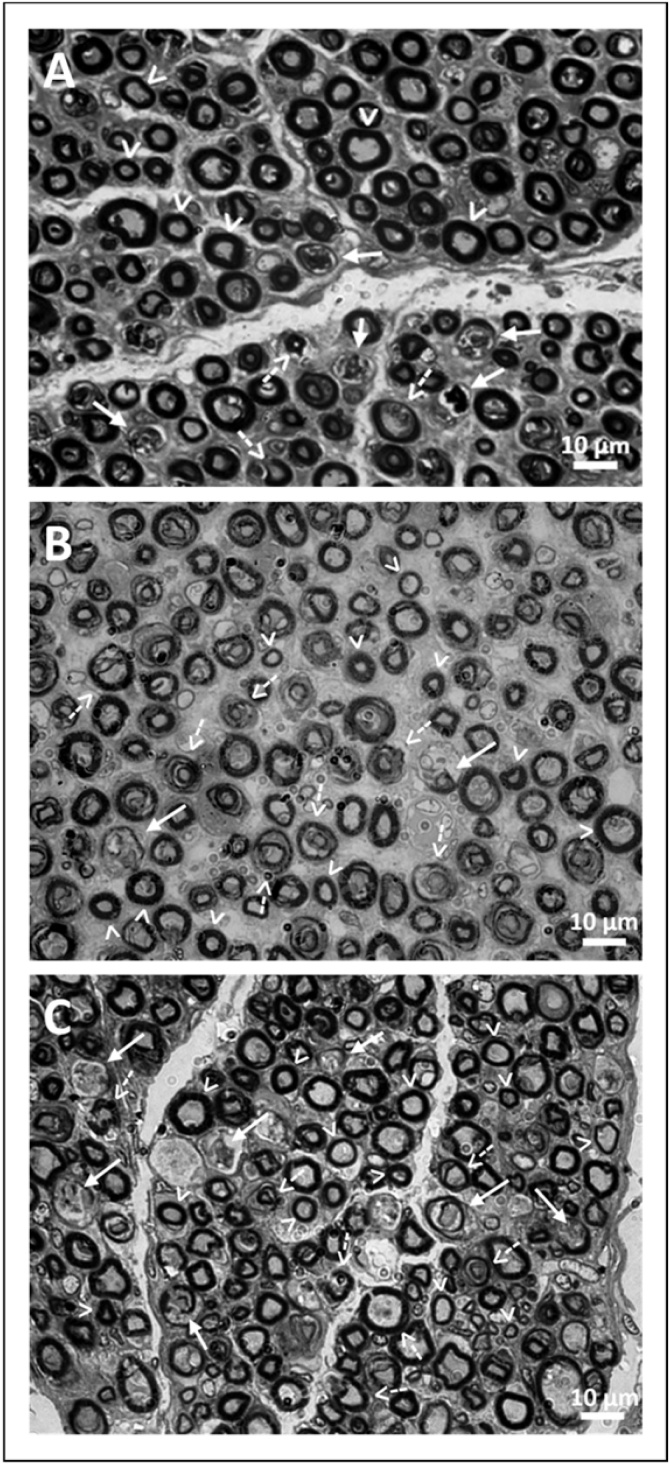
Images showing cross-sections of the mastoid segment of the facial nerve of three cadavers with different age groups at a final magnification of 1,000×. In A, the age group of 60 to 70 years is represented. There is a significant amount of preserved nerve fibers with different areas and thicknesses of myelin sheath. Despite this, nerve fibers in early and advanced stages of neurodegeneration can already be observed. In B, the age group of 70 to 80 years is represented. There is a considerable reduction in the amount of preserved nerve fibers, already indicating a decrease in the area and thickness of the myelin sheath. The neurodegenerated fibers are found in greater quantity when compared to image “A”. In C, the age group over 80 years is represented. In addition to the reduction in the amount of preserved nerve fibers, an increase in fibers with smaller axonal area and myelin sheath thickness is observed. There is an extensive presence of nerve fibers in early and advanced neurodegeneration processes, as evidenced in “B”. Caption: Preserved nerve fiber (arrowhead outline); Nerve fibers in early neurodegeneration processes (dotted arrow); Nerve fiber in advanced neurodegeneration processes (solid arrow).

In this study, microscopic morphometric analysis was performed on 18 facial nerves from cadavers of three different age groups (9 nerves between 60 and 70 years old, 4 nerves in the age group between 71 and 80 years old and 5 nerves older than 80 years old). The nerves presented a monofascicular pattern with nerve fibers surrounded by endoneurial connective tissue evidencing progressive (initial and advanced) processes of neurodegeneration (Fig. 2, Fig. 3).

The cross-sectional area of the nerve decreased with age, being smaller in the group of elderly individuals over 80 years old in relation to the other groups which obtained similar results. Regarding the estimated total number of myelinated fibers and density of myelinated fibers, there was no significant difference between the three age groups.

The area and diameter of myelinated fibers decreased progressively among the groups indicating a progressive reduction with advancing age (p < 0.001; Table 1). Corroborating these findings, the G-ratio increased progressively (p < 0.001 among the 3 groups) and the thickness of the myelin sheath decreased progressively (p < 0.001 among the 3 groups), evidencing a reduction in the myelination of nerve fibers with aging.

**Table 1:** Microscopic morphometric variables analyzed in the facial nerve.

	60–70 years-old (18472 nerve fibers)	71–80 years-old (8734 nerve fibers)	>80 years-old (8797 nerve fibers)	
Variables	Mean ± SD	Mean ± SD	Mean ± SD	p-value
Cross-sectional area(mm^2^)	0.86 ± 0.25^a^	0.84 ± 0.24^a^	0.76± 0.22^b^	<0.001
Estimated number do myelinated fibers	8733 ± 4033	7245 ± 1043	5800 ±1809	0.286
Fiber density (fibers/mm^2^)	61.3 ± 12.6	56.6 ± 21.1	44.6 ± 21.2	0.249
Myelinated fiber area ( μm^2^)	59.7 ± 30.4^c^	54.9 ± 31.1^b^	36.3 ± 27.7^a^	<0.001
Axon Area ( μm^2^)	12.3 ± 11.4^b^	14.5 ± 13.3^b^	10.8 ± 10.5^a^	<0.001
Diameter of myelinate fiber (μm)	8.43 ± 2.25^c^	8.00 ± 2.45^b^	6.30 ± 2.56^a^	<0.001
Axon diameter (μm)	3.68 ± 1.59^b^	3.93 ± 1.75^b^	3.34 ± 1.63^a^	<0.001
G-ratio	0.44 ± 0.15^a^	0.51 ± 0.19^b^	0.53 ± 0.16^c^	<0.001
Myelin sheath thickness (μm)	2.50 ± 1.99^c^	2.06 ±1.41^b^	1.48 ± 0.79^a^	<0.001
Myelinated fiber area (%)	78.6 ± 16.2^b^	70.2 ± 23.6^a^	69.8 ± 20.6^a^	<0.001
Unmyelinated fibers, degenerating tissue and endoneurium area (%)	21.4 ± 16.2^a^	29.8 ± 23.6^b^	30.2 ± 20.6^b^	<0.001

^a,b,c^Equal letters do not differ according to the Tukey posthoc at 5% significance.

n, Number of myelinated fibers analyzed by age group.

Regarding axon measurements, the cross-sectional area and axon diameter presented lower values in the group over 80 years-old (10.8 ± 10.5 μm^2^ and 3.34 ± 1.63 μm; p < 0.001) in relation to the other groups (12.3 ± 11.4 μm^2^ and 3.68 ± 1.59 μm in the group aged 60–70 years and 14.5 ± 13.3 μm^2^ and 3.93 ± 1.75 μm in the group aged 71–80 years). As it was possible to identify in the results, the AST and axonal diameter of elderly people over 80 years old were smaller than the other groups, indicating that the reduction in axonal area occurs later in relation to myelination changes (Table 1).

The percentage of area occupied by myelinated fibers was lower in the groups 71–80 years (70.2% ± 23.6%) and over 80 years (69.8% ± 20.6%) compared with the group 60–70 years (78.6% ± 16.2%; p < 0.001). In association, the percentage of area occupied by unmyelinated fibers, degenerating tissue and endoneurium was higher in the groups over 80 years (30.2% ± 20.6%) and between 71 and 80 years (29.8% ± 23.6%) compared with the group 60–70 years (21.4% ± 16.2%; p < 0.001; Table 1). Although the density of myelinated fibers in the facial nerve did not differ between the groups, when comparing fibers of different sizes, it was found that the percentage of fibers with a smaller cross-sectional area increased with advancing age, while those with a larger area decreased with aging. The findings show that the number of small fibers (< 10 μm^2^) and medium fibers (10–20 μm^2^) increases with aging and the number of large fibers (> 20 μm^2^), decrease with aging (p < 0.001; Table 2).

**Table 2:** Classification of myelinated nerve fibers analyzed according to the cross-sectional area in the different groups studied.

	60–70 years old (n = 2.717)	71–80 years old (n = 1.406)	>80 years old (n = 2.452)	
Variables	n (%)	n (%)	n (%)	p-value
Myelinated fiber area				**<0.001**
<10 μm^2^	45 (1.7)	64 (4.6)	417 (17.0)^a^	
10‒20 μm^2^	122 (4.5)	111 (7.9)	434 (17.7)^a^	
>20 μm^2^	2542 (93.8)^a^	1231 (87.6)^a^	1600 (65.3)	

a Statistically significant association by the residual test adjusted to 5% significance.

## Discussion

Previous studies on facial nerve morphometry provide important information about its anatomical and clinical characteristics. In general, the previous findings were similar to our results and the variability in value may be associated with methodological and individual differences in the subjects analyzed.

Researchers Kondo et al.^15^ performed a morphometric analysis of human facial nerve fibers in the extracranial portion to estimate the total number of myelinated axons and the average cross-sectional area of the myelinated axons. To conduct the study, nerves were collected from 20 cadavers (43–89 years). The results found were an average number of myelinated axons of 6245 ± 860, and the average cross-sectional area of the myelinated axons was 6.31 ± 0.81 μm^2^. According to the authors, the facial nerve showed a significant decrease in the number of myelinated axons with increasing age, which may contribute to delayed recovery from conditions such as Bell's palsy in the elderly. In the study carried out by Hembd et al.,^16^ facial nerves from 36 cadavers (22–97 years), were analyzed, and the results showed that the number of facial nerve axons decreases with age, suggesting that older patients may have reduced nerve function, impacting the success of facial reanimation during surgical procedures.

The characteristics of nerve fibers of the human facial nerve in different age groups were also analyzed by Fujii and Goto.^17^ The nerves were collected from the mastoid portion of the facial nerve. The criteria used for patient selection were age group and availability of cadavers for nerve collection. Eleven cases of individuals of different ages were selected (20–90 years old). Analysis of nerve images showed that the mean number of nerve fibers in the facial nerve was 6254, ranging from 4486 to 7570. It was observed that the mean cross-sectional area of the facial nerve was 0.424 mm^2^ and the mean size of myelinated nerve fibers was 6.23 μm. Although the study had a small number of donors and did not perform statistical analysis, the data indicate a tendency for the number of axons to decrease with aging. In addition, a variation in the cross-sectional area of the facial nerve and in the size of the myelin sheath was observed, suggesting possible structural changes related to aging.

In our study, a mean number of 7260 ± 2295 fibers with no significant differences between the groups of elderly individuals with different age ranges. Recently, Tereshenko et al.^18^ using molecular markers, identified three axonal populations in the distal branches of the facial nerve: motor (ChAT-positive), sympathetic (TH-positive) and afferent (ChAT-negative). The double immunofluorescence technique revealed a total of 11,800 axons in the facial nerve trunk, of which 78% were motor (9,165 cholinergic axons), 21.8% (2,573) sympathetic, and 0.2% (40) afferent. These values are approximately double those reported in previous studies that used different staining techniques: 6,245 ± 860 by Kondo et al.,^15^ 6,684 ± 1,884 by Engelmann et al.,^19^ 5,329 ± 1,376 by Hembd et al.,^16^ 6,254 by Fujii and Goto,^17^ 8,495 by Thurner et al.^20^ indicating that histological processing and staining techniques can influence the morphological results.

The reduction in the average cross-sectional area of the facial nerve found in this study reveals the neurodegenerative effects of aging. Since no significant changes in the number and density of myelinated axons were identified, this reduction can be attributed mainly to the reduction in the cross-sectional area of myelinated axons that occurred with advancing age. Corroborating our findings, a study analyzed, through magnetic resonance imaging, the facial and cochlear nerves of 157 ears belonging to 102 patients aged between 18 and 76 years and identified a significant decrease in the diameter and cross-sectional area of the facial nerve with aging.^21^

Additionally, these changes in cross-sectional area reduction with advancing age may be associated with a reduction in the bony canal through which the facial nerve travels and also with changes in the central neural pathways. In this sense, Kerimzade^22^ identified, through computed tomography exams, that aging causes a decrease in the cross-sectional area of the second portion of the facial canal, showing that the change in the bony canal may be associated with a reduction in the cross-sectional area of the facial nerve.

These findings of our study showed a degenerative pattern related with aging and corroborate with previous research on the impact of aging on the myelin sheath.^21^^,^^23^, ^24^, ^25^, ^26^, ^27^ Several authors point out that structural changes in the facial nerve, related to the reduction of the myelin sheath, can impair sensory and motor functions of the facial muscles, such as proprioception and facial expression.^20^^,^^26^^,^^27^ Thurner et al.^20^ analyzed 20 samples of the human facial nerve and observed an increase in the G-ratio with age (0.76 in the elderly), indicating progressive demyelination. Schröder et al.^26^ analyzed the thickness of the myelin sheath in cranial nerves such as the facial nerve and in peripheral nerves such as the sural and femoral nerves, and found a significant reduction in myelin in older individuals. This structural loss was accompanied by an increase in unmyelinated fibers at more advanced ages, suggesting an ineffective regenerative process.

In addition, as it was possible to identify in our results, the mean of axonal diameter and area, had significant reductions only in the group over 80 years old, indicating that axonal atrophy occurs later in relation to myelination changes, phenomena already documented in previous studies.^28^^,^^29^ Verdú, Butí, and Navarro^28^ observed that, in mice, myelin degenerates more rapidly due to the reduction of proteins essential for its maintenance, such as protein zero and myelin basic protein. On the other hand, axonal atrophy occurs later, associated with decreased axonal transport and failure to maintain the axonal cytoskeleton. Thus, axons can remain functional for longer despite compromised myelin, due to their capacity for regeneration and adaptation. Lu et al.^29^ corroborate this theory by observing the vagus nerve in 30 human cadavers and reporting that myelin loss precedes axonal atrophy. Although the area and perimeter of myelinated axons decrease with age, the total number of axons remains relatively constant until more advanced stages. These studies reinforce our findings and ratify the hypothesis that axonal atrophy occurs later in relation to the deterioration of the myelin sheath. This may help to elucidate the chronology and pathophysiology of axonal neurodegeneration and directly impact the clinical treatment of these patients.

In the present study, a higher percentage of myelinated fibers with a larger cross-sectional area was identified in all groups, i.e., neurons with a probable motor function for facial mimic muscles. As aging progressed, in the comparison between the three groups of elderly individuals, progressive atrophy of myelinated nerve fibers was identified, resulting in a percentage increase in fibers with a smaller cross-sectional area and a percentage reduction in fibers with a larger cross-sectional area.

Previous studies have reported such fiber behaviors and their morphofunctional implications with advancing age. Hunter, Pereira and Keenan^30^ investigated the neuromuscular system in aging and pointed out that the decrease in the diameter of larger fibers, combined with the reduction in myelin thickness, results in impaired motor performance. Bouche^31^ in his review on neuropathies in aging, also pointed out that the loss of myelin and the reduction in fiber diameter, especially Aα fibers, compromise motor and sensory functions, impairing proprioception and mobility in the elderly. Similarly, Ward et al.^32^ concluded in their review that the reduction in the functionality of nerve fibers in the lower limbs of elderly individuals is associated with a decrease in the diameter and thickness of myelin. These studies corroborate our results and suggest that aging is related to a decrease in fiber diameter, particularly larger fibers such as Aα fibers, and as a result, they may lose the ability to perform their functions effectively, impairing sensory and motor functions in the elderly.

Additionally, and corroborating our findings, Verdú et al.,^33^ in a review article, reported changes in myelinated nerve fibers of elderly nerves, including demyelination, deficient remyelination, reduction in axonal diameter, and an increase in degenerating tissue. According to the authors, axonal atrophy in aging has been attributed to a reduction in the expression and transport of cytoskeletal proteins in the peripheral nervous system. Additionally, changes in the responsiveness of axons, Schwann cells, and macrophages also decrease over time, culminating in functional and electrophysiological deficits, including reduced nerve conduction velocity, muscle strength, sensory discrimination, autonomic responses, and endoneurial blood flow. With all this, the capacity for regeneration, although present, becomes less effective with aging.

Our work stands out for its analysis and description of micromorphometric changes in the facial nerve associated with aging, analyzing the nerve in a region of anatomical and surgical relevance, such as the stylomastoid foramen. Micromorphometric changes in the facial nerves associated with age highlight the importance of recognizing that myelin and axonal neurodegeneration is a progressive process that accompanies advancing age and is part of the reality of the elderly. Knowledge of this condition by the surgical and therapeutic team can positively impact the treatment and quality of life of patients with dysfunctions affecting the facial nerve.

## Conclusion

This study described the microscopic morphometry of the mastoid segment of the facial nerve in the region of the stylomastoid foramen in elderly individuals of three different age groups. The estimated total number and density of myelinated nerve fibers of the facial nerve were not altered in the groups of elderly individuals evaluated in this study. There was a progressive reduction in myelination of nerve fibers with a reduction in the percentage of total area occupied by myelinated fibers, accompanied by an increase in the percentage of area occupied by the endoneurium, unmyelinated fibers and degenerative tissue. A reduction in the cross-sectional area of myelinated axons was identified in individuals over 80 years of age, compared to other age groups, showing that these changes occur later in the aging process. In the groups with older age, there was an increase in the percentage of myelinated fibers with a smaller cross-sectional area and a reduction in the percentage of myelinated fibers with a larger cross-sectional area.

## ORCID ID

Mateus Belmonte Macedo: 0000-0001-5195-3643

Adriana Ubirajara Silva Petry: 0000-0002-7756-4328

## Funding

This study was supported by financial assistance from the 10.13039/501100004909UFRGS.

## Data availability statement

The authors declare that all data are available in repository.

## Declaration of competing interest

The authors declare no conflicts of interest.
